# Current Approach to Non-Infectious Pulmonary Complications of Hematopoietic Stem Cell Transplantation

**DOI:** 10.4274/balkanmedj.2017.1635

**Published:** 2018-03-15

**Authors:** Güldane Cengiz Seval, Pervin Topçuoğlu, Taner Demirer

**Affiliations:** 1 Department of Hematology, Ankara University School of Medicine, Cebeci Hospital, Ankara, Turkey

**Keywords:** Hematopoietic stem cell transplantation, pulmonary complications, Bronchiolitis Obliterans syndrome, Idiopathic Pneumonia syndrome

## Abstract

Hematopoietic stem cell transplantation is an established treatment for patients with a wide range of malignant and nonmalignant conditions. Noninfectious pulmonary complications still remain a leading cause of morbidity and mortality in these patients. Treating hematopoietic stem cell transplantation recipients with noninfectious pulmonary complications is still challenging, and the current treatment armamentarium and strategies are not adequate for patients receiving hematopoietic stem cell transplantation. Further trials are needed for a better description of the pathogenesis and the complete diagnostic criteria as well as for the development of effective therapeutic approaches for the management of noninfectious pulmonary complications of the hematopoietic stem cell transplantation. This review outlines the incidence, risk factors, pathogenesis, and clinical spectrum and discusses the current approaches to the management of noninfectious pulmonary complications of Hematopoietic stem cell transplantation.

Hematopoietic stem cell transplantation (HSCT) has been increasingly used for the treatment of a wide range of benign and malignant disorders (1,2,3,4,5). However, HSCT is still limited due to the development of serious complications associated with the occurrence of either acute or chronic graft versus host disease (aGVHD, cGVHD). Advances in the pretransplant conditioning regimens and post-transplant immunosuppression have contributed to improved overall survival (6). Despite these advancements, pulmonary complications still develop in 30%-60% of HSCT recipients and can account for approximately 50% of transplant-related mortality (6,7). The timeline of the primary pulmonary complications following HSCT is shown in Figure 1. Factors that influence the development of pulmonary complications in HSCT include an underlying disease, the age of the patient, previous infections (pretransplant serostatus), the conditioning regimen, current or prior immunosuppressive and radiation treatment, the type of stem cell transplant (autologous/allogeneic), the use of prophylactic antibiotics, and the time passed on the transplant (7,8,9,10,11,12). In recent years, the spectrum of pulmonary complications following HSCT has changed increasingly from infectious to noninfections etiologies with the judicious use of broad-spectrum antimicrobial prophylaxis (13). The approach for the evaluation of pulmonary complications occurring after allogeneic HSCT depends on the post-transplant time interval and the engraftment status of HSCT recipients. Since most of the treatable pulmonary complications in patients are diagnosed noninvasively and frequently via bronchoscopy, a surgical lung biopsy is rarely needed. Restrictive and obstructive ventilatory defects and gas transfer abnormalities have been observed frequently after HSCT (13,14). Several research groups have demonstrated the correlation between pretransplant pulmonary function test (PFT) abnormalities and the risk of respiratory failure in various cohorts (15,16). In a study of 52 young, asymptomatic patients, 23% had restrictive defects with or without impaired gas transfer and 15% had isolated impaired gas transfer before HSCT (13). However, the role of PFT in identifying HSCT recipients at risk for respiratory failure needs validation through larger prospective studies. This review also discusses the PFT findings of pulmonary complications. Noninfectious pulmonary complications still remain a significant problem following HSCT, in both acute and chronic settings. In approximately 50% of cases, no infectious microorganisms are identified in the lungs of affected patients (17). It is important to note that this type of complications is associated with significant morbidity and mortality and poor response to standard treatments. Periengraftment Respiratory Distress syndrome (PERDS), diffuse alveolar hemorrhage (DAH), Idiopathic Pneumonia syndrome (IPS), bronchiolitis obliterans organizing pneumonia (BOOP), and Bronchiolitis Obliterans syndrome (BOS) are all unique subsets of noninfectious complications (Table 1) (18). To our knowledge, there is no standard guideline regarding an approach to the management of pulmonary complications following HSCT. In addition, due to the availability of limited prospective controlled studies assessing the efficacy of different treatments, no standard therapy can be recommended. A suggested algorithmic approach to the management of pulmonary complications after HSCT is outlined in Figure 2. This review focuses on the definition, risk factors, and pathogenesis of the primary noninfectious pulmonary complications in HSCT recipients.

## IDIOPATHIC PNEUMONIA SYNDROME

IPS is an important cause of acute pulmonary complications after HSCT. In 1993, a panel consensus by the National Institutes of Health (NIH) clarified IPS as a widespread alveolar injury following HSCT in the absence of active lower respiratory tract infection or cardiogenic causes (20). Recently, in 2011, the American Thoracic Society updated the diagnostic criteria of IPS (21). From a practical standpoint, the definition of IPS depends on the exclusion of infectious organisms, cardiac failure, acute renal insufficiency, or iatrogenic fluid overload in the setting of lung injury following HSCT. IPS encompasses a heterogeneous entity of disorders that results from common pathological findings of acute interstitial pneumonitis, DAH, PERDS, and Delayed Pulmonary Toxicity syndrome (DPTS); chemotherapy-related lung injury (21). The etiology and pathogenesis of IPS have not been well defined. Various studies have shown that the incidence of IPS ranges from 3% to 15%, and these complications generally occur within 4 months after allogeneic HSCT with myeloablative conditioning (22,23). In 2003, Fukuda et al. (24) found a significantly lower cumulative incidence of IPS in patients at 120 days after nonmyeloablative conditioning (2.2% vs. 8.4%; p=0.003). Once established, however, lung injury was severe and the overall mortality rate was 75%, regardless of therapy, with a 2-week median time to death after the onset of IPS following HSCT (25). A systemic analysis of 20 studies (1090 patients) demonstrated an association between the incidence of IPS and the use of high-dose lung irradiation, high-dose cyclophosphamide, and the addition of busulfan (24,26,27). Data generated from murine models propose that these conditioning agents produce lung epithelial injury followed by excessive requirement and activation of pulmonary macrophages and alloreactive T-lymphocytes (21). The other factors responsible for developing IPS following HSCT include an underlying diagnosis of acute leukemia or Myelodysplastic syndrome, older patient age, lower pretransplant performance status, total body irradiation (TBI), high-grade aGVHD, and positive donor cytomegalovirus serology (21). To date, the impact of the stem cell source (bone marrow vs. peripheral blood vs. umbilical cord blood) on the development of IPS has not been fully sought. Of note, despite the attempts to reduce the risk factors, the overall mortality due to IPS remains substantially high. The clinical manifestations of IPS include dyspnea, dry cough, hypoxemia, and radiographic opacities, which are similar to pneumonia of infectious etiology (20). Bronchoscopy with bronchoalveolar lavage (BAL) or transbronchial biopsy, if there is no contraindication, needs to be performed for the diagnosis of IPS cases. PFT and thorax CT are nonspecific. More than 90% of recipients with IPS have diffuse infiltrates on chest radiography as observed in the infections. Kantrow et al. (28) demonstrated the identification of IPS in 80% of patients by BAL, of whom 4% required lung biopsy and 16% were diagnosed at autopsy. Diffuse alveolar damage, organizing or acute pneumonia, and interstitial lymphocytic inflammation are observed in the lung biopsies of patients with suspected IPS.

The median time of onset of IPS is 21-65 days, ranging from 0 to 1653 days following allogeneic HSCT (21,24,29). Historically, the clinical outcomes among HSCT recipients with IPS have been generally poor, with an estimated overall mortality rate of 74% (range, 60%-80%) (7). The 1-year OS is <15% (28,30). Most of the patients with IPS develop progressive respiratory failure requiring mechanical ventilation, in which the mortality rate exceeds 95% (28). Treating patients with IPS is still challenging since there are no data clarifying the type of the relevant therapy for HSCT recipients. Currently, the only accepted treatment option is supportive care (supplemental oxygen, diuretics) combined with broad-spectrum antimicrobial agents with or without intravenous high-dose glucocorticoids of 1 mg/kg or more (6). Although a small number of reports have shown limited responses to standard therapy, the mortality in IPS cases still remains unacceptably high (14). Advances in supportive care, including the early institution of continuous veno-venous hemofiltration, may help to improve survival in selected patients. Unfortunately, prospective studies addressing the treatment of patients with IPS, including the specific use of corticosteroids, do not exist in the literature (7). Of note, trials with larger sample sizes have demonstrated no benefit of glucocorticoids in the outcome of patients (28,30). The mixed inflammatory alveolar infiltrates described in mice with IPS are associated with an increase in the levels of certain cytokines [e.g., interleukin-6, interleukin-8, tumor necrosis factor alpha (TNFα)] in both lung tissue and BAL fluid (31,32). Based on these results, sequential trials of the combination of a systemic corticosteroid with a TNFα inhibitor (etanercept or infliximab) have been conducted. Recently, a research group from the University of Michigan reported the outcomes of 15 patients with IPS who were treated with glucocorticoids and etanercept (0.4 mg/kg and maximum 25 mg) twice weekly with a maximum of eight doses. In the evaluation of that report, 67% of the patients had a complete response within 3-18 days, and the survival rates at day 28 and day 56 (from the first etanercept dose) were 73% and 60%, respectively (29). Based on these encouraging results, investigators from the University of Pennsylvania conducted a randomized placebo-controlled trial with single-agent corticosteroids or in combination with etanercept; favorable outcome was obtained in the combination group following the onset of IPS (at day 28: 88.2% vs. 36.4%, p<0.001, and at 2 years: 18% vs. 9.1%, p<0.003) (6). The Children’s Oncology Group and the Pediatric Blood and Marrow Transplant Consortium recently completed a phase II, open label trial of etanercept along with glucocorticoids and reported similar response rates that approximate prior series in the preliminary analysis (6). With enormous progress in the understanding of the pathways involved in the development of IPS, treatment of IPS may lead into a new era. Ultimately, the low accrual rates point out the inherent difficulties in conducting definitive trials on HSCT patients with IPS.

## DIFFUSE ALVEOLAR HEMORRHAGE

In 1989, Robbins et al. (33) defined a description of a subset of patients who have diffuse pulmonary infiltrates, fever, hypoxemia, thrombocytopenia, and renal failure occurring within the first few weeks after autologous HSCT for solid malignancy (33,34,35,36,37,38). The primary feature of this syndrome was progressive bloodier return of BAL fluid without frank hemoptysis (39). Most commonly, DAH occurs in the early period after HSCT and develops in 5%-12% of HSCT recipients with a median time to onset of 19 days (range, 5-34 days) in allogeneic recipients (21). However, a higher incidence of DAH has been reported in HSCT recipients with inherited metabolic storage disease, especially mucopolysaccharidosis (40). The pathogenesis of DAH after HSCT is not clearly understood. The presence of neutrophils in the BAL fluid of some HSCT recipients with DAH proposes an inflammatory process. The diagnosis of DAH is based on progressively hemorrhagic return on sequential BAL from at least three separate subsegmental bronchi and the presence of >20% hemosiderin-laden macrophages in BAL fluid, which may require as long as 48-72 h to appear on cytological analysis in the absence of a respiratory tract infection (7,41). Hemosiderin-laden macrophages are also observed in patients with thrombocytopenia or pulmonary hypertension (42). Risk factors for DAH after HSCT include age older than 40 years, TBI, HSCT for solid malignancies, and the presence of high fevers, severe mucositis, neutrophil engraftment, and renal failure (33,43). To date, the effect of conditioning regimens or the stem cell source on the development of DAH remains to be elucidated. Common symptoms are dyspnea (92%), fever (67%), cough (56%), and hemoptysis (15%) (39). Most of the individuals with DAH need intensive care unit admission and mechanical ventilation. Patients with DAH typically have patchy or diffuse infiltrates that start centrally on thorax high-resolution computed tomography (HRCT). Surgical lung biopsies or postmortem examination in DAH shows the proliferative phase of diffuse alveolar damage (33,34,35,36,37,38,39,40,41,42,43,44,45,46,47). Prospective randomized trials regarding the treatment of DAH have not yet been conducted. Based on anecdotal reports and retrospective studies, systemic high-dose glucocorticoids (500-1000 mg/day of methylprednisolone for 3-4 days followed by dose-tapering over 4 weeks) are usually administered to treat DAH (48,49).

In 2006, Wanko et al. (50) determined that high-dose glucocorticoid therapy (>4 mg/kg/day of methylprednisolone equivalent) may have increased efficacy with decreased mortality rates (91% vs. 67%) and the addition of aminocaproic acid may further improve the outcomes. In contrast to this report, Rathi et al. (51) from Duke University showed no significant differences in outcome with or without the addition of aminocaproic acid, regardless of methylprednisolone doses. Similarly, no significant response to corticosteroid treatment was noted in a small retrospective pediatric DAH trial (52). The reported mortality rates in HSCT recipients with DAH range from 70% to 100% (24,53). There is only one report from Mayo Clinic, which demonstrated a low mortality rate of 33% (7). It has to be mentioned that alveolar hemorrhage is a distinct entity with extremely poor outcomes following therapy with corticosteroids.

## PERDS (ENGRAFTMENT SYNDROME)

PERDS occurs early in the post-transplant period. A study from Mayo Clinic first described the clinical entity comprising fever of ≥38.3 °C, radiographic diffuse pulmonary infiltrates, absence of cardiac insufficiency, hypoxemia, and an erythematous maculopapular rash (not attributable to drugs), with no identified infectious etiology (54). PERDS develops within 96 h of neutrophil recovery, with an incidence of 7%-11% (55). This syndrome is more common in autologous HSCT recipients (56,57,58,59,60). The pulmonary manifestations of PERDS are due to noncardiogenic diffuse capillary leak. Chest radiography and chest CT show bilateral ground-glass infiltration and hilar or peribronchial consolidation. Neutrophils may be observed in BAL fluid. The causes of this syndrome are still unclear, but the production of proinflammatory cytokines during engraftment is believed to play a regulatory role (61). Notably, the incidence and severity of this condition may be increased by the use of granulocyte-colony-stimulating factor (GCSF); therefore, discontinuation of GCSF is recommended for HSCT recipients with PERDS (62,63,64,65,66,67). A rapid response to corticosteroids has been postulated in patients with pulmonary involvement, but the mortality rate remains unacceptably high after progressing to respiratory failure (54,56).

## DELAYED PULMONARY TOXICITY SYNDROME

DPTS is defined by the presence of interstitial pneumonitis and fibrosis, which may delay for months to years (2). The incidence of DPTS is 29%-64% in autologous HSCT recipients who have received conditioning regimens containing cyclophosphamide, cisplatin, etoposide, and bischloroethylnitrosurea (BCNU) (68,69). The median time of onset of DPTS is 45 days (range, 21-49 days) after HSCT (21).

Patients with DPTS present with fever, dyspnea, cough, and hypoxemia and have patchy or diffuse mixed reticular infiltrates on the chest radiograph (7). Lung biopsy demonstrates diffuse alveolar damage, interstitial pneumonitis, and thickening of the interstitium with early fibrosis (17). In a series of patients with breast cancer receiving high-dose cyclophosphamide, cisplatin, and BCNU followed by autologous HSCT, >30% decline in diffusing gas transfer was observed in 15-18 weeks following the chemotherapy (68). In addition, 17% improvement in diffusing capacity of carbon monoxide (DLCO) had been demonstrated among symptomatic patients treated with corticosteroids (prednisone 60 mg/day for 2 weeks, followed by a 6-week taper) (68). It has to be mentioned that there are no prospective and randomized trials to establish the efficacy of corticosteroid treatment in this setting. No deaths attributable to DPTS were reported. It is important to note that high incidence, low mortality, and good response to glucocorticoid therapy distinguish this process from IPS.

## BRONCHIOLITIS OBLITERANS SYNDROME

BOS is the most common late noninfectious pulmonary complication following allogeneic HSCT. It was initially defined in the 1980s as a histologic entity of small airway inflammation with intraluminal fibrosis (70,71). BOS is a clinical term described based on PFT abnormalities without histological confirmation (72). The incidence of BOS after allogeneic HSCT varies widely from 1.7% to 26%, and, consequently, the nonuniform diagnostic criteria were used to define the condition (73). In 2005, the NIH Consensus Statement on the diagnosis and staging of cGVHD consisted of strict diagnostic criteria for BOS and subsequently recommended modifications that improved the identification of HSCT recipients with BOS (74). The development of BOS is closely associated with the presence of cGVHD. Other frequently observed risk factors for BOS include methotrexate use, serum immunoglobulin deficiency (especially immunoglobulins G and A), prior occurrence of aGVHD, older recipient (>20 years) and donor age, pretransplant low forced expiratory volume in 1 s (FEV1)/forced vital capacity (FVC) ratio (<0.7), and respiratory viral infections within the first 100 days after HSCT (72). In the multivariate analysis reported by the IBMTR (International Blood and Marrow Transplant Registry), busulfan-based conditioning regimen, peripheral blood stem cell transplantation, duration from the diagnosis of leukemia to HSCT (>14 months), female donor to male recipient, history of interstitial pneumonitis, and an event of moderate-to-severe aGVHD have been found as risk factors for BOS (75,76,77,78). The pathogenesis of BOS after HSCT has not been very well defined. Several hypotheses have been suggested; however, none of them have a satisfactory explanation for the BOS pathogenesis. The strong association between BOS and cGVHD proposes alloimmunological injury to host bronchiolar epithelial cells (75). Indeed, some investigators suggest that BOS is a manifestation of cGVHD. There are also alternative theories such as viral infections, recurrent aspiration due to GVHD-associated esophagitis, lung injury precipitated by the conditioning chemoradiotherapy, abnormal local defense mechanisms in the lung, and impaired mucociliary transport (79). In 2002, Hauber et al. (80) compared the results of BAL fluid analysis of 11 HSCT recipients with pulmonary complications (six had IPS and/or BOS) with those of 11 healthy volunteers. They found significantly higher levels of TNFα and interleukin-18 in the BAL fluid analysis of the patient cohort compared with those of the controls (80). The median onset time of BOS is approximately 1 year (range, 3 months to 10 years) (81,82). In an IBMTR report, the median interval from transplant to diagnosis of BOS was found to be 431 days (range, 65-2444 days) (82). The common respiratory symptoms are dry cough (60%-100%), progressive dyspnea (50%-70%), and wheezing (40%) (83,84). Unlike BOOP, fever is rare in BOS. A total of 20% of patients remain asymptomatic despite having evidence of abnormal PFT findings (83). More than 33% of recipients with cGVHD have evidence of airflow obstruction, and these patients should be followed up closely (75). Spirometry is the primary procedure used to diagnose and follow up HSCT recipients with BOS. PFT usually shows new-onset airflow obstruction with a decrease in FEV1 and FEV1/FVC ratio. Chest radiography is often normal or may represent hyperinflation (83). Expiratory air trapping, mosaic perfusion, bronchial dilatation, and bronchial wall thickening are generally observed on thorax HRCT (85). Bronchoscopy with BAL in HSCT recipients with BOS is nonspecific with the presence of neutrophilic and/or lymphocytic inflammation (86). BAL is primarily performed to rule out infectious etiologies. Transbronchial lung biopsy is generally not recommended because of the involvement of respiratory and membranous bronchioles in this condition. Surgical lung biopsy by video-assisted thoracoscopic surgery is required for histological confirmation. Lung biopsies reveal small airway involvement with fibrogenic deposition (79). A recent study established that histological BOS diagnosis was not superior to clinical diagnosis depending on clinical symptoms, PFT abnormalities, and radiographic signs beyond the time of biopsy (87). The following diagnostic criteria for BOS were recently reported by an NIH workshop: (i) FEV1/FVC <0.7 and FEV1 <75% of predicted value; (ii) HRCT of chest (with inspiratory and expiratory views) showing spaces of air trapping or small airway thickening or bronchiectasis, residual volume of PFT >120% of predicted value, or histological confirmation of constrictive bronchiolitis; and (iii) absence of infection in the respiratory tract documented by clinical symptoms, radiological procedures, or microbiological cultures (88). Another significant determination of the NIH Consensus Development Project is to form a scoring system of pulmonary cGVHD based on symptoms and PFT findings. There are no prospective, randomized trials addressing the standard treatment of BOS. Based on small, uncontrolled trials and expert opinions, high-dose systemic corticosteroids and augmented immunosuppression are administered for treatment (89,90). Prednisolone 1-1.5 mg/kg/day (up to 100 mg/day) for 2-6 weeks is suggested, and if the respiratory status remains stable, the dose is tapered every 2 weeks for 6-12 months. Immunosuppression with cyclosporine A or azathioprine is initiated when there is no response to corticosteroid therapy within 1 month (91). In addition, azathioprine doses are 2-3 mg/kg/day (maximum 200 mg/day), and cyclosporine A dose should be adjusted according to serum levels for 3-12 months. Extracorporeal photodynamic (ECP) has been proven to be an effective treatment option for cGVHD. The published reports on the effectiveness of ECP for HSCT recipients with BOS are even less compelling. The Regensburg conference recommended ECP as a frontline therapy choice for BOS (79). However, there are only three reports encouraging the benefits of this therapy in pulmonary manifestations of cGVHD. Lucid et al. (92) conducted the only study using ECP in patients with BOS following HSCT in 2011. This retrospective analysis reported some degree of stabilization in FEV1 in six of nine patients with BOS, comparing FEV1 values prior to and during ECP treatment (92).

BOS following HSCT is a progressive disorder that may lead to irreversible airflow obstruction. Intensive treatments resulting in an improvement of pulmonary functions are noted in only 8% and 20% of cases (7). The goal of the treatment of BOS must be stabilization and prevention of further drops in FEV1. The published mortality rates vary from 14% to 100% (mean, 61%) (7). The major causes of death are progressive respiratory failure and opportunistic lung infections. Rapid deterioration of FEV1 (more than 10% per year), which is refractory to first-line therapy, recipient age >60 years, progressive cGVHD, and prior respiratory viral infections are associated with a worse prognosis (72). With clarification of the specific pathways in BOS, anti-inflammatory and immunomodulatory therapies targeting these pathways may be developed for the future management of HSCT recipients with BOS.

## BRONCHIOLITIS OBLITERANS ORGANIZING PNEUMONIA

BOOP was first described in the early 1990s and presents more like pneumonia than airway disease within the first 2-6 months following HSCT (93). It is characterized by an extensive infiltration of granulation tissue within alveolar ducts and alveoli, consisting of fibroblasts and a matrix of loose connective tissue. In contrast to BOS, BOOP is exclusively an alveolar disorder; there is no prominent bronchiolar damage (94). Recently, to avoid confusion with BOS terminology, BOOP has been renamed as cryptogenic organizing pneumonia (95). Even though the pathogenesis of BOOP after HSCT is not clearly understood, published reports have postulated that alloimmune injury is the primary triggering factor for its development. In murine models, BOOP occurs after retrovirus infections in which T cells and T helper 1-derived cytokines, including interferon-α, play an important role in the development of this process (96). Although a consensus on the identification criteria is lacking, the incidence of BOOP is 0.9%-10.3% (61). Freudenberger et al. (97) reviewed the data of 5.340 patients who underwent allogeneic HSCT, and 49 cases (0.9%) of histologic BOOP were diagnosed. A report from Mayo Clinic analyzed the incidence of BOOP among HSCT recipients and reported that 1.6% and 10.3% of patients who underwent HSCT from matched sibling donors and unrelated donors respectively developed BOOP (98). The risk factors for BOOP include HLA disparity, HSCT from a female donor to a male recipient, and the use of peripheral blood as a stem cell source. Notably, an association between GVHD and the subsequent occurrence of BOOP has been reported (72). In the literature, the development of BOOP has also been defined as somehow a rejection of the lung by the donor’s stem cells (7). BOOP usually presents with fever, nonproductive cough, and dyspnea. A mild-to-moderate restrictive defect (FVC <80%, FEV1/FVC ≥80%), commonly decreased DLCO, and in contrast to BOS, normal expiratory flow are observed in the PFT (6). The radiologic findings in BOOP include peripheral patchy air space consolidation termed as “fluffy,” ground-glass and nodular opacities. Lee et al. (99) reported the radiographic patterns in 43 HSCT recipients with biopsy-proven BOOP. Consolidation was the most common pattern in 79% of cases; ground-glass attenuation and nodular opacities were noted in 60% and 30%, respectively (99). Bronchoscopy and BAL fluid are useful to exclude pulmonary infection and for the demonstration of BOOP. Lymphocytosis with a decreased CD4+/CD8+ ratio was revealed in the cytological analysis of BAL fluid (72). Transbronchial or surgical lung biopsy procedures are considered as the gold standard for diagnosis. The histologic hallmark of BOOP is the presence of patchy intraluminal fibrosis resembling granulation tissue in distal airways extending to the alveolar ducts and peribronchial alveolar spaces (100). Although there is no standard treatment for HSCT recipients with BOOP, corticosteroid is the mainstay of treatment. Based on limited retrospective reports, it has been shown that approximately 80% of patients respond favorably to systemic corticosteroid therapy (7). The initial dose of corticosteroid treatment is prednisone 0.75-1.5 mg/kg/day for 1-3 months and 40 mg for 3 months, followed by 10-20 mg for a total of 1 year. Reported relapse rate varies from 9% to 58%; therefore, prolonged treatment courses have been recommended (101). Recently, Radzikowska et al. (102) compared the outcome of corticosteroid and clarithromycin treatment in patients with biopsy-proven BOOP. Complete remission was achieved in 88% of patients treated with clarithromycin and in all patients treated with corticosteroids. Of note, recurrence was observed more frequently in the corticosteroid group (54.5% vs. 10%; p<0.0001) (102). Freudenberger et al. (97) reported that BOOP following HSCT resolved in 57% of patients and remained stable in 21% of cases. Progression of disorder was observed in 11 (22%) patients despite corticosteroid therapy, with initial doses ranging from 1 mg/kg/day to 2 g/day. Of the 11 patients, 8 (73%) died of respiratory failure attributed to BOOP (97). It is important to note that more clinical trials should be conducted to understand the dose and duration of corticosteroid therapy.

## PULMONARY VENO-OCCLUSIVE DISEASE

Pulmonary veno-occlusive disease (PVOD) occurs rarely and has a late onset (after the first 100 days) after allogeneic HSCT (39). PVOD is identified by intimal proliferation and fibrosis of the pulmonary venules and small veins that result in progressive vascular obstruction with high pulmonary and capillary pressure (103). Patients with PVOD present with dyspnea and fatigue within post-transplant 3-4 months. PFT shows a mild restrictive ventilation defect and a decreased DLCO. Chest radiographs and CT reveal a pleural effusion, diffuse or mosaic ground-glass attenuation, and nodular opacities. CT pulmonary angiography excludes the evidence of thrombi as a cause of pulmonary hypertension. The diagnosis of PVOD cannot be made unless right-sided heart catheterization demonstrates findings with elevated pulmonary artery pressure (≥25 mmHg) and a normal pulmonary artery wedge pressure (<15 mmHg). The triad of pulmonary artery hypertension, radiographic signs of pulmonary edema, and a normal pulmonary artery occlusion pressure strongly proposes the diagnosis of PVOD (103).

Treatment-associated risks such as pulmonary edema and respiratory failure are higher with vasodilators, which must be initiated under close observation. Hackman et al. (104) reported the efficacy of high-dose corticosteroid therapy (methylprednisolone 2.0 mg/kg/day) in a series of two patients with PVOD.

## PULMONARY CYTOLYTIC THROMBI

Pulmonary cytolytic thrombi (PCT) is an unusual pulmonary complication of HSCT (105). The etiology of new-onset PCT after HSCT is unknown. The median time of onset of PCT is 72 days (range, 8-343 days). PCT occurs particularly in children with GVHD and consequently has been believed to be a manifestation of acute and chronic GVHD (105). It should be suspected in HSCT recipients with fever and numerous peripheral pulmonary nodules on chest CT. The prognostic procedure of choice is surgical lung biopsy showing necrotic, basophilic thromboembolism with entrapped monocytes (106). The clinical presentations improve within 1-2 weeks, and radiographic findings disappear over weeks to months. There is no proven therapy for PCT. Recently, investigators from the University of Minnesota reported the outcome of 14 HSCT recipients with PCT who had received 1-2 mg/kg/day of prednisone (or equivalent methylprednisolone dosing) until the resolution of pulmonary symptoms (typically 1-2 weeks, followed by a rapid taper over 2-4 weeks) with a 3-year OS of 71% (105).

Pulmonary complications are frequently observed after HSCT and remain a leading cause of morbidity and mortality. Effective prophylaxis of infections with antibiotics is changing the scene of pulmonary complications from infectious to noninfectious etiologies. The pathogenesis and diagnostic criteria of these complications have not yet been clearly defined. It is important to note that treatment of HSCT recipients with noninfectious pulmonary complications is still challenging, and the current treatment armamentarium and strategies are not adequate. Therefore, further trials are needed for a better description of the pathogenesis and the complete diagnostic criteria as well as for the development of effective therapeutic approaches for the management of noninfectious pulmonary complications of HSCT recipients.

## Figures and Tables

**Table 1:**
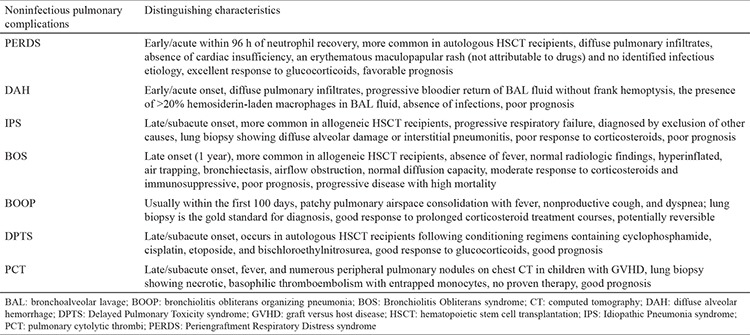
The distinguishing characteristics of the noninfectious pulmonary complications in hematopoietic stem cell transplantation

**Figure 1:**
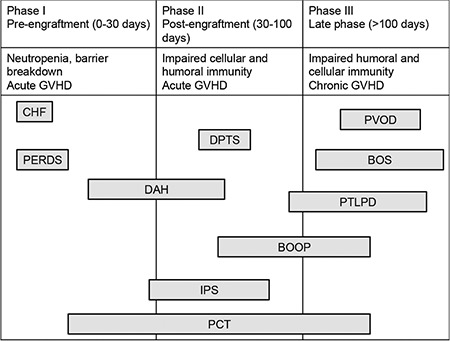
The timeline of the primary pulmonary complications following [Chi et al. (19)]. *BOS: Bronchiolitis Obliterans syndrome; BOOP: bronchiolitis obliterans organizing pneumonia; CHF: congestive heart failure; DAH: diffuse alveolar hemorrhage; DPTS: Delayed Pulmonary Toxicity syndrome; GVHD: graft versus host disease IPS: Idiopathic Pneumonia syndrome; PCT: pulmonary cytolytic thrombi; PERDS: Periengraftment Respiratory Distress syndrome; PTLPD: post-transplant lymphoproliferative disorder; PVOD: pulmonary veno-occlusive disease*

**Figure 2:**
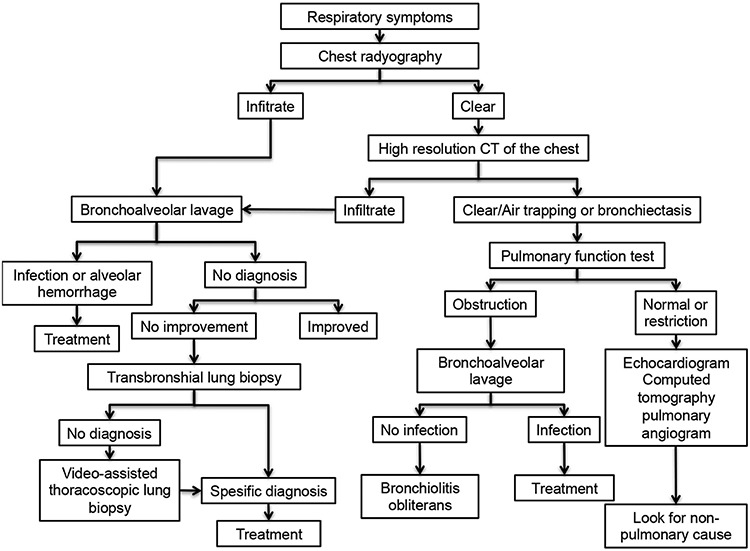
An algorithmic approach to the management of pulmonary complications after hematopoietic stem cell transplantation [Afessa et al. (7)].
